# Expression of BNIP3 in invasive breast cancer: correlations with the hypoxic response and clinicopathological features

**DOI:** 10.1186/1471-2407-9-175

**Published:** 2009-06-09

**Authors:** Esther A Koop, Theo van Laar, Dick F van Wichen, Roel A de Weger, Elsken van der Wall, Paul J van Diest

**Affiliations:** 1 Department of Pathology, University Medical Center, Utrecht, The Netherlands; 2 Division of Internal Medicine and Dermatology, Medical Center, Utrecht, The Netherlands

## Abstract

**Background:**

Bcl-2/adenovirus E1B 19 kDa-interacting protein 3 (BNIP3) is a pro-apoptotic member of the Bcl-2 family induced under hypoxia. Low or absent expression has recently been described in human tumors, including gastrointestinal tumors, resulting in poor prognosis. Little is known about BNIP3 expression in invasive breast cancer. The aim of the present study was to investigate the expression of BNIP3 in invasive breast cancer at the mRNA and protein level in correlation with the hypoxic response and clinicopathological features.

**Methods:**

In 40 cases of invasive breast cancer, BNIP3 mRNA *in situ *hybridization was performed on frozen sections with a digoxigenin labeled anti-BNIP3 probe. Paraffin embedded sections of the same specimens were used to determine protein expression of BNIP3, Hypoxia Inducible Factor 1 alpha (HIF-1α) and its downstream targets Glucose Transporter 1 (Glut-1) and Carbonic Anhydrase (CAIX) by immunohistochemistry.

**Results:**

BNIP3 mRNA was expressed in 16/40 (40%) of the cases and correlated with BNIP3 protein expression (p = 0.0218). Neither BNIP3 protein nor mRNA expression correlated with expression of HIF-1α expression or its downstream targets. Tumors which showed loss of expression of BNIP3 had significantly more often lymph node metastases (82% vs 39%, p = 0.010) and showed a higher mitotic activity index (p = 0.027). BNIP3 protein expression was often nuclear in normal breast, but cytoplasmic in tumor cells.

**Conclusion:**

BNIP3 expression is lost in a significant portion of invasive breast cancers, which is correlated with poor prognostic features such as positive lymph node status and high proliferation, but not with the hypoxic response.

## Background

Bcl-2/adenovirus E1B 19 kDa-interacting protein3 (BNIP3) is a member of the Bcl-2 family. The common feature among all Bcl-2 family members is the presence of one or several Bcl-2 homology domains, of which four different domains have been identified. The proteins are divided according to these homology domains into pro- and anti-apototic proteins [1]. In the anti-apoptotic group, all four homology domains are present. In BNIP3 and many of the other pro-apoptotic proteins, only the BH3 domain is present and BNIP3 therefore belongs to the BH3-only family. BNIP3 protein is localized on the outside of the mitochondrial membrane. Upon stimulation it integrates into the mitochondrial membrane, leading to permeabilization of the transition pore and a decrease of the mitochondrial membrane potential. This results in chromatin condensation and DNA-fragmentation and finally apoptosis [2,3]. BNIP3-induced cell death is caspase-independent and does not induce cytochrome c release [2]. BNIP3 has also implicated in the induction of autophagy/mitophagy [4,5].

Hypoxia has been described as an important inducer of BNIP3 expression. The transcription factor Hypoxia Inducible Factor (HIF-1α) is the key regulator of the hypoxic response. Under normoxic conditions HIF-1α is hydroxylated, followed by binding to Von Hippel Lindau (VHL) protein and proteosomal degradation. Under hypoxic conditions this hydroxylation does not occur, and in case of VHL mutations proteosomal degradation is prohibited, leading to HIF-1α stabilization, heterodimerization with its constitutively expressed partner HIF-1β and translocation to the nucleus, where it transactivates hypoxia responsive genes by binding to a Hypoxia-Responsive-Element (HRE) in their promoters. Among the target genes of HIF-1α are genes involved in glycolysis, angiogenesis and apoptosis [6]. Previous studies indicated that HIF-1α enhances the expression of BNIP3 [6-8] and that levels of BNIP3 protein usually parallel mRNA levels [8]. Hypoxia-induced autophagy via BNIP3 has been claimed to be a survival mechanism promoting tumor progression [9].

Decreased levels of BNIP3 mRNA and protein have been found in pancreatic cancer [10-12], colorectal and gastric cancer [13], and hematopoietic malignancies [14]. On the other hand, increased levels of BNIP3 protein have been described in non-small cell lung cancer, where high expression of BNIP3 protein was linked with a poor prognosis [15,16]. In prostate cancer, there was a significant correlation between cytoplasmic BNIP3 expression and Gleason score, age, and Glut1, and between nuclear BNIP3 expression and HIF-1α [17].

Only a few studies on BNIP3 expression in breast cancer have been published. One study showed high BNIP3 mRNA expression in breast tumors compared to normal tissue, especially in peri-necrotic tumor region [18]. BNIP3 mRNA was also strongly expressed in ductal carcinoma *in situ *(DCIS) and correlated with a high grade phenotype and presence of invasive disease [19]. Another study using an experimental mouse model showed that expression of BNIP3 protein was inversely correlated with the ability of tumor cells to metastasize. Knockdown of BNIP3 increased tumor size and induced metastases [20].

A more recent study [21] suggested that upregulation of BNIP3 protein expression plays a role in breast tumor progression. This study showed an increased disease-free survival in patients with ER-positive BNIP3 nuclear-positive invasive tumors, but no associations with cytoplasmic BNIP3. Furthermore they found no association between HIF-1α and BNIP3.

In the present study we have investigated the expression of BNIP3 in invasive breast cancer at the mRNA and protein level in correlation with the hypoxic response and clinicopathological features. For the hypoxic response, we examined HIF-1α as the key regulator and two of its target genes, Glucose Transporter 1 (Glut-1) and Carbonic Anhydrase 9 (CAIX).

## Methods

### Human breast tumor samples

Forty specimens of invasive breast carcinoma, surgically resected between 2004 and 2006, were derived from the archives of the Department of Pathology, University Medical Center Utrecht, The Netherlands. Anonymous use of leftover tumor material is part of the standard agreement with patients in our hospital [22], so no explicit ethical approval or informed consent was needed according to Dutch law. The Science Committee of the Department of Pathology of the University Medical Center Utrecht approved the use of frozen section material. For all specimens, formalin-fixed, paraffin embedded material as well as fresh-frozen tissue was available. Thirty cancers were of ductal type, 3 were lobular, 3 ducto-lobular, 3 mucinous and 1 was a medullary carcinoma. All carcinomas were histologically graded according the Bloom and Richardson (B&R) grade [23,24]. Necrosis was noted on the same sections, and the mitotic activity index was assessed as a marker of proliferation as previously described [25]. Estrogen (ER) and progesterone receptor (PR) status were assessed by standard immunohistochemistry.

### *In situ *hybridization

The probe for BNIP3 mRNA *in situ *hybridization was synthesized by amplification of a 496 bp BNIP3 fragment from human placental cDNA. Primers were selected through Primer Select (version 4.00 1993–1999, DNA Star Inc., Madison, USA) resulting in the forward primer sequence 5'GCCCGGGATGCAGGAGGAGA and the reverse primer sequence 5'GAGCAGCAGAGATGGAAGGAAAAC. Primers were purchased from Eurogentec S.A, Seraing, Belgium. PCR conditions were as follows: one denaturing cycle at 95°C for 5 min, followed by 30 cycles of 94°C for 30 s, 60°C for 30 s, and 72°C for 1 min before a final extension step at 72°C for 10 min.

These primers resulted in a primary PCR product of 496 bp, which was sequenced to assure a 100% nucleotide match with BNIP3 mRNA. A re-PCR was performed to label the BNIP3 PCR-product with digoxigenine, by replacing part of the dTTP in the PCR mix by Dig-11-dUTP (Boehringer-Mannheim GmbH, Mannheim, Germany). Digoxigenine-labeled PCR-products were purified and used as probe for *in situ *hybridization.

Fresh frozen sections of 8 μm were fixed in 4% formaldehyde for 1 h at room temperature. After washing, endogenous peroxidase was blocked with 0.3% H_2_O_2 _in phosphate buffered saline (PBS) for 20 min. The cells were made accessible for probes by protein K (0.1 μg/ml in PBS) at 37°C and Triton X100 (0.005% in PBS) treatment. Sections were subsequently dehydrated, air dried and incubated with 25 μl hybridization mix (30% formamide, Tris-EDTA buffer, 20× SSC, yeast tRNA, herring sperm DNA and denatured probe) for 10 min at 47°C on a hot plate to stretch the mRNA, followed by 18 h incubation at 37°C in a humid atmosphere.

After hybridization, slides were washed and the hybridized probe was detected by Polyclonal Rabbit Fab-2 antidigoxigenine/HRP (1:4000 DakoCytomation, Glostrup, Denmark) in PBS/1% bovine serum albumin and visualized by Tyramide FITC (1:50 in amplification diluent (Perkin Elmer, Boston, USA). DNA in the nucleus was visualized with 4 μg/ml propidiumiodide (Sigma Chemicals, St. Louis, USA) in PBS. After washing, the slides were embedded in Fluorescent Mounting Medium (Dakocytomation).

### Assessment of *in situ *hybridization

The pVHL defective cell line RCC10 [26], derived from a sporadic renal clear cell cancer, was used as a positive control for BNIP3 expression, and a pVHL wild type cell line (20) and a slide without the denatured probe were used as negative controls. The results with these control cell lines were checked by RT-PCR with both BNIP3-specific primers and primers for Glyceraldehyde-3-phosphate dehydrogenase (GAPDH) as household gene.

Hybridized tissues were analyzed using a confocal laser scanning microscope (Leica TCS, Rijswijk, The Netherlands). Stromal macrophages and lymphocytes served as internal control for BNIP3 expression, as they showed a similar strong mRNA expression in all slides, as confirmed by immunohydrido double staining for CD68 respectively CD3 (data not shown), whereas tumor BNIP3 signal intensity differed between the different samples. Samples were binary scored as negative/weak or positive.

### Immunohistochemistry

Serial sections of 4 μm of paraffin embedded material of each specimen were deparaffinized and rehydrated in graded alcohol. Endogenous peroxidase activity was blocked by 0.3% hydrogen peroxide in PBS for 20 min. Antigen retrieval was performed by boiling the slides 20 min in 10 mM citrate buffer (pH 6.0) for BNIP3, CAIX and Glut-1, and in EDTA buffer (pH 9.0) for HIF-1α immunohistochemistry. To avoid non-specific binding of BNIP3, slides were pre-incubated with 10% normal swine serum in PBS. Thereafter slides were incubated with antibodies against BNIP3 (Rabbit polyclonal BNIP3, Biocarta Europe GmbH, Hamburg, Germany, dilution 1:1000), CAIX (Rabbit polyclonal, Abcam, Cambridge, UK, dilution 1:1000), HIF-1α (Mouse monoclonal, BD Transduction Laboratories, Lexington, USA dilution 1:50) or Glut-1 (Dako, dilution 1:200).

Incubation with anti-BNIP3 and anti-HIF-1α was done overnight at 4°C, and with anti-CAIX and anti-Glut-1 at room temperature for 1 h.

After incubation, slides were washed and incubated with Powervision (Immunologic, Duiven, The Netherlands), final staining was developed by adding 3,3 diaminobenzidine tetrahydrochloric acid in PBS (1.5% H_2_O_2_) and slides were counterstained with haematoxylin.

A renal clear cell carcinoma (CAIX, Glut-1, BNIP3), an invasive breast cancer (HIF-1α,) and placental tissue (BNIP3) were used as controls for immunohistochemistry [27]. Negative controls were obtained by omission of the primary antibodies.

### Quantification of immunohistochemistry

All slides were scored independently by two pathologists (EAK/PJvD). Samples were binary scored as negative/weak or positive. When possible, both the tumor and the benign tissue were scored. Glut-1 as well as CAIX staining were scored as positive when membrane staining was seen. For HIF-1α the percentage of positive nuclei was scored as well as the topography: perinecrotic, diffuse or a combination of both. Only homogenously and darkly stained nuclei were considered and a percentage of ≥ 1% positive nuclei was considered positive in line with previous studies. [28].

### Statistical analysis

Fisher's exact test (GraphPad Instat, version 3.01, Graphpad Software Inc., San Diego, USA) was used to test the correlation between BNIP3 *in situ *hybridization and immunohistochemistry and the other immunohistochemical and clinicopathological features. For HIF-1α, the traditional threshold of 5% was used. Two-sided p-values < 0.05 were considered significant.

## Results

### *In situ *hybridization

**Figure 1:**
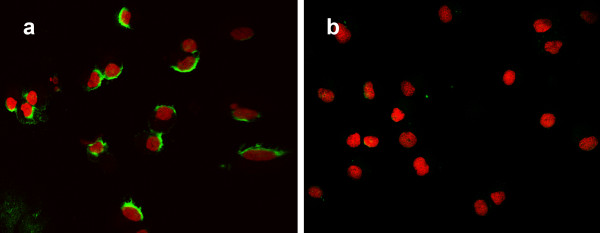
***In situ *hybridization for BNIP3-mRNA**. BNIP3-mRNA (green) in control cells. Nuclei are visualized with Propidiumiodide (red). (a) BNIP3 mRNA expression in the pVHL defective cell line RCC10. (b) BNIP3 mRNA expression in a pVHL wild type cell line.

**Figure 2:**
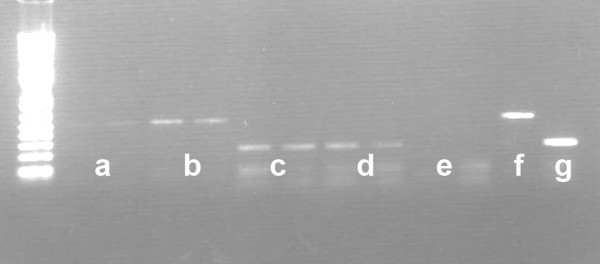
**RT-PCR results in duplicate**. A pVHL wild type cell line with (a) BNIP3 amplicon compared to a pVHL defective cell line with (b) BNIP3 amplicon. As control lane (c) shows a pVHL wild type cell line with GAPDH amplicon, compared to a pVHL defective cell line (d) with GAPDH amplicon. Lane (e) shows negative H_2_O control, and (f) and (g) show positive controls with placental cDNA with BNIP3 amplicon (f) and with GAPDH amplicon (g).

The positive control RCC10 cell line showed an intense signal in the cytoplasm (Fig. 1a), whereas cells of the pVHL wild type cell line showed no or very low BNIP3-mRNA signal (Fig. 1b). RT-PCR for BNIP3 band resulted in a much more intense band in the pVHL defective cell line compared to the pVHL intact cell line (Fig 2). As expected this effect was not seen for the household gene *GAPDH*.

**Figure 3:**
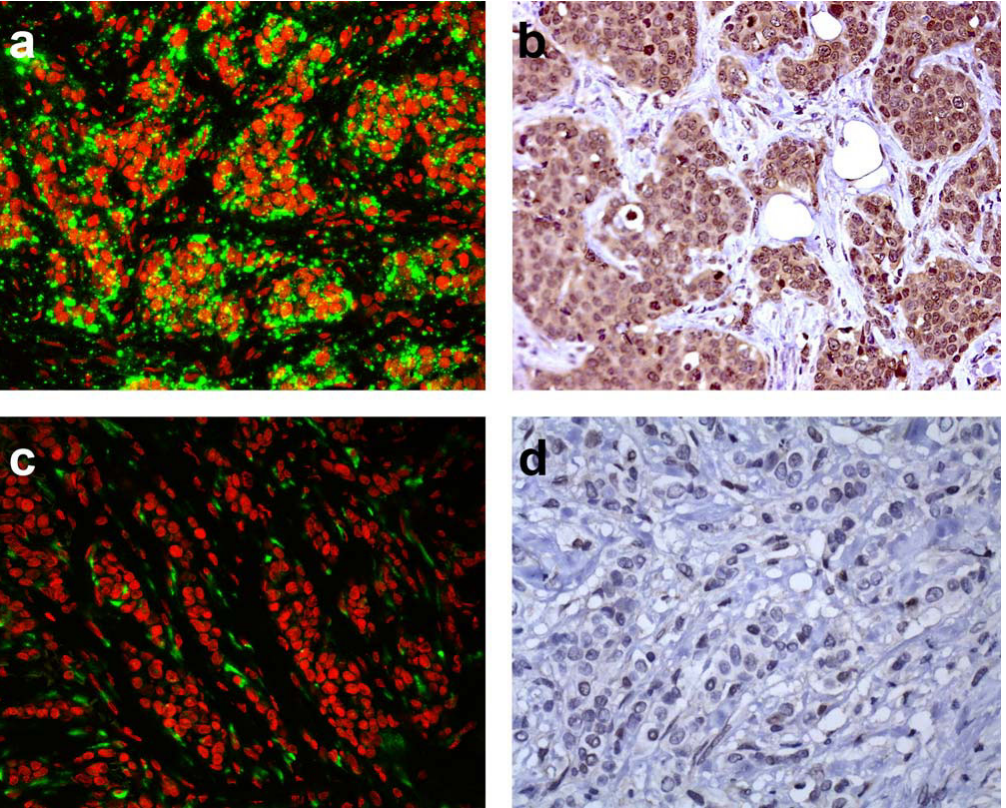
***In situ *hybridization and immunohistochemistry for BNIP3 (original magnification 20×)**. (a) BNIP3 mRNA expression in a positive tumor and (b) strong BNIP3 protein expression in the same tumor. (c) BNIP3 mRNA negative tumor with presence of positive stromal macrophages as internal controls and (d) absence of BNIP3 protein expression in the same tumor.

Sixteen of the 40 breast carcinomas (40%) were positive for BNIP3 mRNA. In all cases the signal was strong throughout the tumor, and tumor cell staining was often granular (Fig 3a), compared to the more diffuse signal in macrophages. In the remaining 60% of cases, tumor cells were negative or had only a very weak cytoplasmic signal (Fig 3c), whereas the macrophages still had their diffuse strong signal.

### Immunohistochemistry

Twenty-three out of 40 tumors (57%) were positive for BNIP3 protein (Fig 3b). Most of these tumors had a diffusely positive cytoplasmic signal throughout the tumor. The remaining 17 cases were negative or had a very weak expression (Fig 3d). Expression of BNIP3 protein correlated with BNIP3 mRNA expression (p = 0.022).

**Figure 4:**
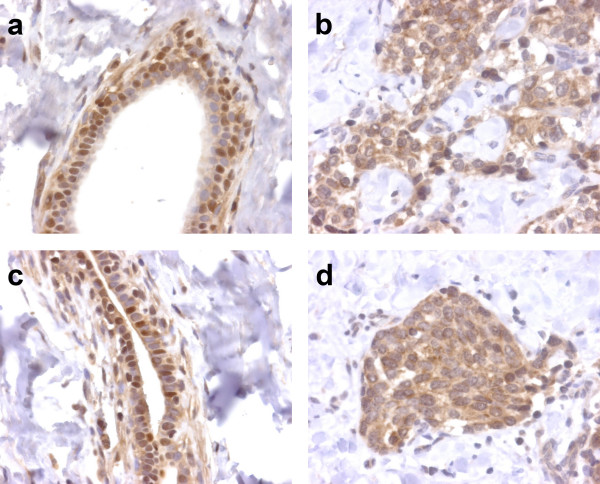
Immunohistochemistry for BNIP3 in 2 cases of invasive breast cancer (original magnification 40×) showing nuclear staining in adjacent normal breast tissue (a and c), while tumor cells of the same specimens show only cytoplasmic expression (b and d).

In 35 cases there was sufficient adjacent normal breast tissue that could be examined. In 8 of these 35 cases, the normal breast tissue showed nuclear staining whereas this was rarely seen in tumor cells (Fig. 4). When tumor cells were strongly positive, the adjacent normal tissue showed a clearly lower signal in 13/20 (65%) of cases, whereas the normal tissue showed a strong signal similar to the tumor cells in 7/20 (35%) of cases. In 6/15 (40%) of cases where tumor cells had a very low signal or no BNIP3 expression, the normal breast tissue showed more BNIP3 expression than the tumor cells.

**Figure 5:**
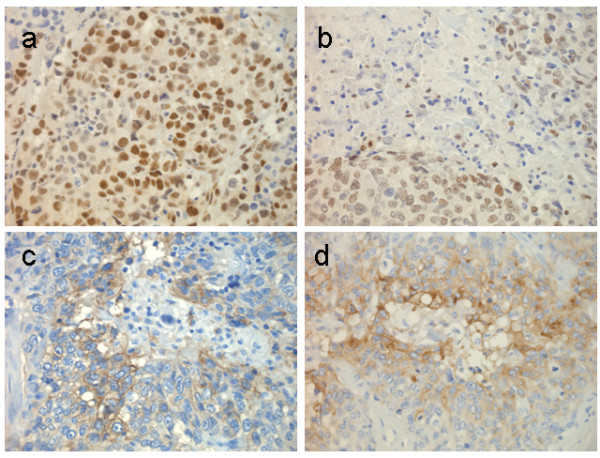
**Representative examples of immunohistochemical staining for (a) diffuse HIF-1α, (b) perinecrotic HIF-1α, (c) CAIX and (d) Glut-1 in an invasive breast cancer (b, c, d same case)**. Original magnification 40×.

HIF-1α was positive in 24/40 tumors (60%), 8 of them in a perinecrotic and 16 in a diffuse pattern. CAIX and Glut-1 membrane staining were seen in 16/40 (40%) and 15/40 (38%) of cancers, respectively. Figure 5 shows some representative examples. There was a significant correlation between expression of Glut-1 and CAIX (p = 0.018). CAIX and Glut-1 expression was absent in normal tissue surrounding the tumors.

Positive HIF-1α staining was typically associated with necrosis. Of the 24 HIF-1α positive tumors, 12 showed CAIX (p = 0.006) and 12 showed Glut-1 expression (p = 0.017), especially those with necrosis. Most diffusely positive HIF-1α cases were Glut-1 negative (69%); 50% were negative for CAIX.

Neither BNIP3 mRNA nor protein status showed significant relations with HIF-1α status (Table 1). BNIP3 was however, more often although not significantly expressed in diffusely HIF-1α positive cases (10/16, 62%) than in perinecrotic HIF-1α positive cases (2/6, 33%) (p = 0.353). BNIP3 protein and mRNA expression failed to correlate with CAIX or Glut-1 expression (Table 1).

**Table 1:** Association between BNIP3 mRNA and protein expression and the hypoxia response proteins in invasive breast cancer (n = 40).

		BNIP3 mRNA			BNIP3 protein		
	Grouping	Low	High	p-value	Low	High	p-value

**HIF-1α**	0	12	4	0.188	5	11	0.332
			
	≥ 1%	12	12		12	12	

**Glut-1**	Negative/cytoplasmic staining	17	8	0.204	10	15	0.749
			
	Membrane staining	7	8		7	8	

**CAIX**	Negative/cytoplasmic staining	16	8	0.338	9	15	0.522
			
	Membrane staining	8	8		8	8	

### Clinicopathological features

As shown in Table 2, tumors negative for BNIP3 protein, had a significantly higher frequency of axillary lymph node metastases than tumors with BNIP3 expression (82% vs 39%, p = 0.010). For BNIP3 mRNA the same inverse association was seen (p = 0.001). The mitotic index showed a similar inverse association with BNIP3 protein expression (p = 0.027). Tumor size, histological grading, necrosis, ER and PR were not significantly associated with BNIP3 protein expression (see Table 2), as for BNIP3 mRNA.

**Table 2:** Association of BNIP3 mRNA and protein expression with clinicopathological features in invasive breast cancer (n = 40).

	BNIP3 mRNA			BNIP3 protein		
Feature*	Low	High	p-value	Low	High	p-value
**Lymph node status**						
**Negative**	5	12	0.001	3	14	0.009
**Positive**	19	4		15	9	
**MAI (2 mm**^2^**)**						
**1–12**	9	9	0.335	4	14	0.027
**≥ 13**	15	7		13	9	
**ER**						
**Negative**	7	6	0.733	5	8	1.000
**Positive**	17	10		12	15	
**PR**						
**Negative**	12	7	0.755	10	9	0.337
**Positive**	12	9		7	14	
**Tumor size**						
**0–2 cm**	7	6	0.733	3	10	0.103
**> 2 cm**	17	10		14	13	
**Histological grade**						
**I/II**	11	10	0.349	6	15	0.108
**III**	13	6		12	8	
**Necrosis**						
**Negative**	17	9	0.500	10	16	0.520
**Positive**	7	7		7	7	

* MAI: mitotic activity index, ER: estrogen receptor, PR: progesterone receptor.

## Discussion

The aim of the present study was to investigate the expression of BNIP3 in invasive breast cancer at the mRNA and protein level in correlation with the hypoxic response and clinicopathological features. Twenty three out of 40 tumors (57%) were positive for BNIP3 protein. The remaining 17 cases were negative or had a very weak expression. Expression of BNIP3 protein correlated with BNIP3 mRNA expression (p = 0.022).

To test the hypoxic response in invasive breast carcinomas in relation to BNIP3, we also examined the expression of HIF-1α and its target genes Glut-1 and CAIX [29-31]. CAIX and Glut-1 showed the expected positive correlation with HIF-1α (p = 0.006, p = 0.017, respectively), especially with perinecrotic HIF-1α [31], indicating that the study group is large enough to study the hypoxic response. Of all tumors tested, 16 showed BNIP3 mRNA expression. Twelve (75%) of these also had HIF-1α expression. The remaining 25% had no HIF-1α, but did show BNIP3 mRNA. Thereby, the correlation between HIF-1α protein and BNIP3 was not significant at the mRNA or protein level. In a previous study [19], perinecrotic expression of BNIP3 mRNA and protein was reported in DCIS, but it was not mentioned whether this was also seen in invasive cancer, which we did not find. Also in another recent study [21] there was no association between HIF-1α and BNIP3. There are therefore no firm clues that BNIP3 expression in invasive breast cancer is hypoxia dependent. Induction of BNIP3 may however also occur through non-hypoxic stimulation. The zinc-finger protein PLAGL2 is for example able to increase BNIP3 levels, but is independent of the hypoxia-responsive element in the BNIP3-promoter [32]. In this current study twenty four tumors (60%) had no BNIP3 mRNA expression. A likely explanation for the absence of BNIP3 mRNA could be methylation of the BNIP3-promoter, which would result in silencing of the gene. This has been shown in pancreatic adenocarcinoma where the promoter of *BNIP3 *turned out to be methylated in all BNIP3-negative pancreatic cancer cell lines and in eight out of 10 pancreatic adenocarcinoma samples [12]. Impaired hypoxia induced BNIP3 expression has also been detected in other malignancies. Sixty six percent of the primary colorectal and 49% of the gastric cancers showed BNIP3 methylation [13,14]. Also in haematological malignancies methylation of the BNIP3 gene has been described, although less frequently [14]. BNIP3 promoter methylation in breast cancer has however not yet been described.

Another mechanism of mediation of BNIP3 activity may be intranuclear sequestration of BNIP3 protein, that has been reported in glial cells of the normal brain and in glioblastoma multiforme [33]. In our study, we did not find nuclear BNIP3 protein in tumors cells, but in 23% of cases in adjacent normal breast tissue. This fits with the concept of intranuclear sequestration of BNIP3 in normal breast tissue, but the exact mechanism remains unclear and should be part of further research.

By examining the relationship between BNIP3 expression and clinicopathological features, we found a highly significant inverse correlation between BNIP3 expression and presence of lymph node metastases, both on the protein level and mRNA level, this in contrast to a previous study [21]. Of the 24 BNIP3 mRNA negative tumors, 79% had axillary lymph node metastases, whereas this was only 25% in the BNIP3 mRNA positive group. Furthermore, BNIP3 expression correlated with the mitotic index, which is known to be a strong prognostic indicator in breast cancer [25,34]. Survival analysis is not yet possible because of the short follow-up time, but 3 patients had distant metastases in the lung, bones or brain at the time of completing this study. In all three cases there was no BNIP3 mRNA. All this implies that BNIP3 expression could be a prognostic indicator in breast cancer.

## Conclusion

BNIP3 expression is lost in a significant proportion of invasive breast cancers, and loss of BNIP3 expression correlated with poor prognostic features such as positive lymph node status and high proliferation, but not with the hypoxic response. BNIP3 inactivation may therefore be involved in breast carcinogenesis and deserves to be studied as a potential prognostic indicator in breast cancer.

## Competing interests

The authors declare that they have no competing interests.

## Authors' contributions

EAK carried out the *in situ *hybridization experiments and performed immunohistochemistry, performed the statistical analysis, and drafted the manuscript. TL participated in the design of the study, designed primers for *in situ *hybridization. DFW helped to perform the *in situ *hybridization and immunohistochemistry experiments. RAW participated in the design of the study and helped to set up *in situ *hybridization protocols. EW participated in the design of the study, selected patients and provided clinicopathological data. PJD conceived of the study, and participated in its design and coordination, and supervised statistics. All authors read and approved the final manuscript.

## Pre-publication history

The pre-publication history for this paper can be accessed here:

http://www.biomedcentral.com/1471-2407/9/175/prepub

## References

[B1] Reed JC Bcl-2 family proteins Oncogene 1998 17 25 3225 3236 10.1038/sj.onc.1202591 9916985

[B2] Velde C Vande Cizeau J Dubik D Alimonti J Brown T Israels S Hakem R Greenberg AH BNIP3 and genetic control of necrosis-like cell death through the mitochondrial permeability transition pore Mol Cell Biol 2000 20 15 5454 5468 10891486 10.1128/MCB.20.15.5454-5468.2000 PMC85997

[B3] Imazu T Shimizu S Tagami S Matsushima M Nakamura Y Miki T Okuyama A Tsujimoto Y Bcl-2/E1B 19 kDa-interacting protein 3-like protein (Bnip3L) interacts with bcl-2/Bcl-xL and induces apoptosis by altering mitochondrial membrane permeability Oncogene 1999 18 32 4523 4529 10.1038/sj.onc.1202722 10467396

[B4] Zhang J Ney PA Role of BNIP3 and NIX in cell death, autophagy, and mitophagy Cell Death Differ 2009 in press 10.1038/cdd.2009.16 PMC2768230 19229244

[B5] Azad MB Chen Y Henson ES Cizeau J McMillan-Ward E Israels SJ Gibson SB Hypoxia induces autophagic cell death in apoptosis-competent cells through a mechanism involving BNIP3 Autophagy 2008 4 2 195 204 18059169 10.4161/auto.5278 PMC3164855

[B6] Greijer AE Groep P van der Kemming D Shvarts A Semenza GL Meijer GA Wiel MA van de Belien JA van Diest PJ Wall E van der Up-regulation of gene expression by hypoxia is mediated predominantly by hypoxia-inducible factor 1 (HIF-1) J Pathol 2005 206 3 291 304 10.1002/path.1778 15906272

[B7] Bruick RK Expression of the gene encoding the proapoptotic Nip3 protein is induced by hypoxia Proc Natl Acad Sci USA 2000 97 16 9082 9087 10922063 10.1073/pnas.97.16.9082 PMC16825

[B8] Guo K Searfoss G Krolikowski D Pagnoni M Franks C Clark K Yu KT Jaye M Ivashchenko Y Hypoxia induces the expression of the pro-apoptotic gene BNIP3 Cell Death Differ 2001 8 4 367 376 10.1038/sj.cdd.4400810 11550088

[B9] Bellot G Garcia-Medina R Gounon P Chiche J Roux D Pouysségur J Mazure NM Hypoxia-induced autophagy is mediated through HIF-induction of BNIP3 and BNIP3L via their BH3-domains Mol Cell Biol 2009 29 10 2570 2581 10.1128/MCB.00166-09 19273585 PMC2682037

[B10] Abe T Toyota M Suzuki H Murai M Akino K Ueno M Nojima M Yawata A Miyakawa H Suga T Upregulation of BNIP3 by 5-aza-2'-deoxycytidine sensitizes pancreatic cancer cells to hypoxia-mediated cell death J Gastroenterol 2005 40 5 504 510 10.1007/s00535-005-1576-1 15942716

[B11] Erkan M Kleeff J Esposito I Giese T Ketterer K Buchler MW Giese NA Friess H Loss of BNIP3 expression is a late event in pancreatic cancer contributing to chemoresistance and worsened prognosis Oncogene 2005 24 27 4421 4432 10.1038/sj.onc.1208642 15856026

[B12] Okami J Simeone DM Logsdon CD Silencing of the hypoxia-inducible cell death protein BNIP3 in pancreatic cancer Cancer Res 2004 64 15 5338 5346 10.1158/0008-5472.CAN-04-0089 15289340

[B13] Murai M Toyota M Suzuki H Satoh A Sasaki Y Akino K Ueno M Takahashi F Kusano M Mita H Aberrant methylation and silencing of the BNIP3 gene in colorectal and gastric cancer Clin Cancer Res 2005 11 3 1021 1027 15709167

[B14] Murai M Toyota M Satoh A Suzuki H Akino K Mita H Sasaki Y Ishida T Shen L Garcia-Manero G Aberrant DNA methylation associated with silencing BNIP3 gene expression in haematopoietic tumours Br J Cancer 2005 92 6 1165 1172 10.1038/sj.bjc.6602422 15756280 PMC2361956

[B15] Giatromanolaki A Koukourakis MI Sowter HM Sivridis E Gibson S Gatter KC Harris AL BNIP3 expression is linked with hypoxia-regulated protein expression and with poor prognosis in non-small cell lung cancer Clin Cancer Res 2004 10 16 5566 5571 10.1158/1078-0432.CCR-04-0076 15328198

[B16] Mellor HR Harris AL The role of the hypoxia-inducible BH3-only proteins BNIP3 and BNIP3L in cancer Cancer Metastasis Rev 2007 26 3–4 553 566 10.1007/s10555-007-9080-0 17805942

[B17] Shaida N Launchbury R Boddy JL Jones C Campo L Turley H Kanga S Banham AH Malone PR Harris AL Fox SB Expression of BNIP3 correlates with hypoxia-inducible factor (HIF)-1alpha, HIF-2alpha and the androgen receptor in prostate cancer and is regulated directly by hypoxia but not androgens in cell lines Prostate 2008 68 3 336 343 10.1002/pros.20707 18163427

[B18] Sowter HM Ratcliffe PJ Watson P Greenberg AH Harris AL HIF-1-dependent regulation of hypoxic induction of the cell death factors BNIP3 and NIX in human tumors Cancer Res 2001 61 18 6669 6673 11559532

[B19] Sowter HM Ferguson M Pym C Watson P Fox SB Han C Harris AL Expression of the cell death genes BNip3 and NIX in ductal carcinoma in situ of the breast; correlation of BNip3 levels with necrosis and grade J Pathol 2003 201 4 573 580 10.1002/path.1486 14648660

[B20] Manka D Spicer Z Millhorn DE Bcl-2/adenovirus E1B 19 kDa interacting protein-3 knockdown enables growth of breast cancer metastases in the lung, liver, and bone Cancer Res 2005 65 24 11689 11693 10.1158/0008-5472.CAN-05-3091 16357180

[B21] Tan EY Campo L Han C Turley H Pezzella F Gatter KC Harris AL Fox SB BNIP3 as a progression marker in primary human breast cancer; opposing functions in in situ versus invasive cancer Clin Cancer Res 2007 13 2 Pt 1 467 474 10.1158/1078-0432.CCR-06-1466 17255267

[B22] van Diest PJ No consent should be needed for using leftover body material for scientific purposes. For Bmj 2002 325 7365 648 651 10.1136/bmj.325.7365.648 12242180

[B23] Elston CW Ellis IO Pathological prognostic factors in breast cancer. I. The value of histological grade in breast cancer: experience from a large study with long-term follow-up Histopathology 1991 19 5 403 410 10.1111/j.1365-2559.1991.tb00229.x 1757079

[B24] Elston CW Ellis IO Pathological prognostic factors in breast cancer. I. The value of histological grade in breast cancer: experience from a large study with long-term follow-up. C. W. Elston & I. O. Ellis. Histopathology 1991; 19; 403–410 Histopathology 2002 41 3A 151 10.1046/j.1365-2559.2002.14691.x 12405945

[B25] van Diest PJ Baak JP Matze-Cok P Wisse-Brekelmans EC van Galen CM Kurver PH Bellot SM Fijnheer J van Gorp LH Kwee WS Reproducibility of mitosis counting in 2,469 breast cancer specimens: results from the Multicenter Morphometric Mammary Carcinoma Project Hum Pathol 1992 23 6 603 607 10.1016/0046-8177(92)90313-R 1592381

[B26] Esteban MA Tran MG Harten SK Hill P Castellanos MC Chandra A Raval R O'Brien TS Maxwell PH Regulation of E-cadherin expression by VHL and hypoxia-inducible factor Cancer Res 2006 66 7 3567 3575 10.1158/0008-5472.CAN-05-2670 16585181

[B27] Stepan H L C Purz S Hockel M Horn L-C Placental localization and expression of the celldeath factors BNip3 and Nix in preeclampsie, intrauterine growth retardation and HELLP syndrome European Journal of Obstetrics & Gynecology and Reproductive Biology 2005 122 172 176 10.1016/j.ejogrb.2005.01.017 16219518

[B28] Bos R Groep P van der Greijer AE Shvarts A Meijer S Pinedo HM Semenza GL van Diest PJ Wall E van der Levels of hypoxia-inducible factor-1alpha independently predict prognosis in patients with lymph node negative breast carcinoma Cancer 2003 97 6 1573 1581 10.1002/cncr.11246 12627523

[B29] Chen C Pore N Behrooz A Ismail-Beigi F Maity A Regulation of glut1 mRNA by hypoxia-inducible factor-1. Interaction between H-ras and hypoxia J Biol Chem 2001 276 12 9519 9525 10.1074/jbc.M010144200 11120745

[B30] Wykoff CC Beasley NJ Watson PH Turner KJ Pastorek J Sibtain A Wilson GD Turley H Talks KL Maxwell PH Hypoxia-inducible expression of tumor-associated carbonic anhydrases Cancer Res 2000 60 24 7075 7083 11156414

[B31] Vleugel MM Greijer AE Shvarts A Groep P van der van Berkel M Aarbodem Y van Tinteren H Harris AL van Diest PJ Wall E van der Differential prognostic impact of hypoxia induced and diffuse HIF-1alpha expression in invasive breast cancer J Clin Pathol 2005 58 2 172 177 15677538 10.1136/jcp.2004.019885 PMC1770566

[B32] Mizutani A Furukawa T Adachi Y Ikehara S Taketani S A zinc-finger protein, PLAGL2, induces the expression of a proapoptotic protein Nip3, leading to cellular apoptosis J Biol Chem 2002 277 18 15851 15858 10.1074/jbc.M111431200 11832486

[B33] Burton TR Henson ES Baijal P Eisenstat DD Gibson SB The pro-cell death Bcl-2 family member, BNIP3, is localized to the nucleus of human glial cells: Implications for glioblastoma multiforme tumor cell survival under hypoxia Int J Cancer 2006 118 7 1660 1669 10.1002/ijc.21547 16217754 PMC3158801

[B34] van Diest PJ Baak JP The morphometric prognostic index is the strongest prognosticator in premenopausal lymph node-negative and lymph node-positive breast cancer patients Hum Pathol 1991 22 4 326 330 10.1016/0046-8177(91)90080-9 2050366

